# Supplementary material for: From sequence to enzyme mechanism using multi-label machine learning
Source: BMC Bioinformatics. 2014 May 19;15:150. doi: 10.1186/1471-2105-15-150 (PMC4229970; doi:10.1186/1471-2105-15-150)
Supplement: Additional file 2 — Java code of ml2db. Additional file ml2db_code.tar.gz contains the Java source code to run the multi-label machine learning experiments and save the results to database. The code’s Javadoc is included. [file 1471-2105-15-150-S2.zip › additional file 2/ml2db/ml2db/doc/overview-tree.html]

Class Hierarchy


JavaScript is disabled on your browser.


- Overview
- Package
- Class
- Use
- Tree
- Deprecated
- Index
- Help

- Prev
- Next

- Frames
- No Frames

- All Classes

# Hierarchy For All Packages

Package Hierarchies:

- main.ml2db,
- main.ml2db.al,
- main.ml2db.al.database,
- main.ml2db.al.learning,
- main.ml2db.al.utility,
- main.ml2db.al.utility.confidence,
- main.ml2db.al.utility.globaldistance,
- main.ml2db.al.utility.hybrid,
- main.ml2db.al.utility.neighbours,
- main.ml2db.al.utility.pickers,
- main.ml2db.al.utility.pickers.byfrequency,
- main.ml2db.graphviz,
- main.ml2db.mulan.attributesfiltered,
- main.ml2db.mulan.classifier.lazy,
- main.ml2db.mulan.learn,
- main.ml2db.mulan.learn.crosseval,
- main.ml2db.mulan.learn.evaluation,
- main.ml2db.mulan.learn.traintest,
- main.ml2db.mulan.measuremetrics,
- main.ml2db.mulan.mulanarff,
- main.ml2db.mulan.mulanarff.database,
- main.ml2db.mulan.predict,
- main.ml2db.weka,
- test.ml2db,
- test.ml2db.al,
- test.ml2db.al.utility.confidence,
- test.ml2db.al.utility.globaldistance,
- test.ml2db.al.utility.hybrid,
- test.ml2db.al.utility.neighbourhood,
- test.ml2db.al.utility.pickers,
- test.ml2db.alltests,
- test.ml2db.dataharness,
- test.ml2db.graphviz,
- test.ml2db.mulan.attributesfilter,
- test.ml2db.mulan.classifier.lazy,
- test.ml2db.mulan.learn,
- test.ml2db.mulan.learn.database,
- test.ml2db.mulan.learn.traintest,
- test.ml2db.mulan.measuremetrics,
- test.ml2db.mulan.mulanarff,
- test.ml2db.mulan.mulanarff.database,
- test.ml2db.mulan.predict,
- test.ml2db.weka

## Class Hierarchy

- java.lang.Object
  - weka.core.AbstractInstance (implements weka.core.Instance, weka.core.RevisionHandler, java.io.Serializable)
    - weka.core.SparseInstance
      - main.ml2db.weka.IdentifiedSparseInstance
      - main.ml2db.mulan.mulanarff.IdInstance (implements java.lang.Comparable<T>)
        - main.ml2db.al.utility.neighbours.Neighbourhood
  - java.util.AbstractMap<K,V> (implements java.util.Map<K,V>)
    - java.util.TreeMap<K,V> (implements java.lang.Cloneable, java.util.NavigableMap<K,V>, java.io.Serializable)
      - uk.ac.standrews.utils.main.maputils.MapOfMaps
        - main.ml2db.weka.InstAttValueMap
  - test.ml2db.alltests.AllActiveLearningTests
  - test.ml2db.alltests.AllArffTests
  - test.ml2db.alltests.AllDataTests
  - test.ml2db.alltests.AllExternalPredictionsEvaluationTests
  - test.ml2db.alltests.AllGraphVizTests
  - test.ml2db.alltests.AllMulanArffTests
  - test.ml2db.alltests.AllMulanLearningTests
  - test.ml2db.alltests.AllMulanPredictionTests
  - test.ml2db.alltests.AllMulanTests
  - test.ml2db.alltests.AllPreliminaryTests
  - test.ml2db.alltests.AllTests
  - test.ml2db.alltests.AllTrainTestsTests
  - main.ml2db.mulan.measuremetrics.AnnotationsReader
  - test.ml2db.mulan.attributesfilter.ArffGeneratorForFilterTests
  - junit.framework.Assert
    - junit.framework.TestCase (implements junit.framework.Test)
      - test.ml2db.al.ActiveLearningDatabaseTest
      - test.ml2db.al.ActiveLearningTest
      - test.ml2db.al.AlExperimenterTest
      - test.ml2db.mulan.measuremetrics.AnnotationsReaderTest
      - test.ml2db.dataharness.ArffPropsFilesTest
      - test.ml2db.dataharness.ArffPropsOneTest
      - test.ml2db.dataharness.ArffPropsQueriesOneTest
      - test.ml2db.dataharness.ArffPropsQueriesTest
      - test.ml2db.dataharness.ArffPropsQueriesTwoTest
      - test.ml2db.weka.ArffPropsTableTest
      - test.ml2db.dataharness.ArffPropsTwoTest
      - test.ml2db.weka.ArffTest
      - test.ml2db.mulan.attributesfilter.AttFiltArffTest
      - test.ml2db.mulan.attributesfilter.AttFilterTest
      - test.ml2db.weka.AttributeFactoryTest
      - test.ml2db.al.utility.pickers.AttSetByFrequencyPickerTest
      - test.ml2db.mulan.classifier.lazy.BRkNNALTest
      - test.ml2db.al.utility.confidence.ConfidenceCalculatorTest
      - test.ml2db.al.utility.confidence.ConfidenceMetricTest
      - test.ml2db.mulan.learn.CrossEvaluatorTest
      - test.ml2db.dataharness.DatabaseTest
      - test.ml2db.weka.DataSetCheckerTest
      - test.ml2db.weka.DataSetDbLoaderTest
      - test.ml2db.weka.DataSetGeneratorTest
      - test.ml2db.weka.DataSetManagerTest
      - test.ml2db.weka.DataSetWriterTest
      - test.ml2db.dataharness.DataTableTest
        - test.ml2db.dataharness.DataTableFourTest
        - test.ml2db.dataharness.DataTableOneTest
        - test.ml2db.dataharness.DataTableThreeTest
        - test.ml2db.dataharness.DataTableTwoTest
      - test.ml2db.al.utility.globaldistance.DistancesCalculatorTest
      - test.ml2db.al.utility.globaldistance.DistancesStorageTest
      - test.ml2db.al.utility.globaldistance.DistancesStoreTest
      - test.ml2db.mulan.learn.traintest.EvaluationMetricsTest
      - test.ml2db.mulan.learn.EvaluationParametersTest
      - test.ml2db.mulan.learn.EvaluationResultsTableTest
      - test.ml2db.mulan.learn.ExperimenterTest
      - test.ml2db.al.utility.globaldistance.GlobalDistanceMetricTest
      - test.ml2db.al.utility.hybrid.GlobalDistanceWithConfidenceMetricTest
      - test.ml2db.graphviz.GraphVizArffColouredTest
      - test.ml2db.graphviz.GraphVizArffTest
      - test.ml2db.al.GraphVizPrinterTest
      - test.ml2db.graphviz.GraphVizTest
      - test.ml2db.mulan.mulanarff.IdInstanceTest
      - test.ml2db.mulan.mulanarff.IdMultiLabelInstancesTest
      - test.ml2db.weka.InstancesFillerTest
      - test.ml2db.weka.InstAttValueMapTest
      - test.ml2db.al.utility.pickers.LabelsByFreqPickerTest
      - test.ml2db.al.utility.neighbourhood.LinearNNSearchSkipInstanceItselfTest
      - test.ml2db.mulan.mulanarff.database.MulanArffRecordTest
      - test.ml2db.mulan.mulanarff.MulanArffTest
      - test.ml2db.mulan.mulanarff.MulanDataSetDbLoaderTest
      - test.ml2db.mulan.mulanarff.MulanDataSetGeneratorTest
      - test.ml2db.mulan.mulanarff.MulanDataSetManagerTest
      - test.ml2db.mulan.mulanarff.MulanDataSetWriterTest
      - test.ml2db.mulan.learn.database.MulanDbWriterTest
      - test.ml2db.mulan.mulanarff.MulanInstancesFillerTest
      - test.ml2db.mulan.learn.MulanLearnerTest
      - test.ml2db.al.utility.neighbourhood.NeighbourhoodExperimentDetailsTest
      - test.ml2db.al.utility.neighbourhood.NeighbourhoodNetworkTest
      - test.ml2db.mulan.measuremetrics.PredictionsPerformanceMeasurerTest
      - test.ml2db.mulan.predict.PredictionsSaverTest
      - test.ml2db.al.utility.pickers.RandomAttributePickerTest
      - test.ml2db.al.utility.pickers.RandomAttributeSetPickerTest
      - test.ml2db.mulan.learn.traintest.ResultCheckerTest
      - test.ml2db.mulan.learn.database.ResultsFormatterTest
      - test.ml2db.mulan.mulanarff.SerializerTest
      - test.ml2db.mulan.learn.traintest.TrainTestEvaluatorTest
      - test.ml2db.mulan.learn.traintest.TrainTestExperimenterTest
      - test.ml2db.weka.WekaTest
  - test.ml2db.mulan.attributesfilter.AttFiltDataSetGeneratorTest
  - main.ml2db.weka.AttributeFactory
    - main.ml2db.mulan.mulanarff.MulanAttributeFactory
  - main.ml2db.mulan.mulanarff.AttributeUtils
  - main.ml2db.al.utility.confidence.ConfidenceCalculator
  - main.ml2db.al.utility.confidence.ConfidencesStorage
  - main.ml2db.mulan.learn.crosseval.CrossExperimenter
    - main.ml2db.mulan.learn.traintest.TrainTestExperimenter
      - main.ml2db.al.learning.AlExperimenter
      - main.ml2db.mulan.learn.traintest.TrainTestExperimenterSerialized
  - main.ml2db.mulan.learn.crosseval.CrossInputManager
    - main.ml2db.mulan.learn.traintest.TrainTestInputManager
  - test.ml2db.dataharness.DataOne
  - main.ml2db.weka.DataSetChecker
  - main.ml2db.weka.DataSetDbLoader
    - main.ml2db.mulan.mulanarff.MulanDataSetDbLoader
  - main.ml2db.weka.DataSetGenerator
    - main.ml2db.mulan.mulanarff.MulanDataSetGenerator
  - main.ml2db.weka.DataSetWriter
  - test.ml2db.dataharness.DataTwo
  - uk.ac.standrews.utils.main.database.DbManaged
    - main.ml2db.al.database.AlDataSaver
    - uk.ac.standrews.utils.main.database.DbCreator
      - main.ml2db.al.database.AlDbCreator
  - test.ml2db.alltests.DbManagerForTest
  - main.ml2db.DbManagers (implements java.io.Serializable)
  - test.ml2db.al.utility.globaldistance.DistanceExperimentDetailsTest
  - main.ml2db.al.utility.globaldistance.DistancesCalculator (implements java.io.Serializable)
  - main.ml2db.al.utility.globaldistance.DistancesStorage (implements java.io.Serializable)
  - main.ml2db.mulan.learn.evaluation.EvaluationParameters
  - mulan.evaluation.Evaluator
    - main.ml2db.mulan.learn.evaluation.EvaluatorNoAUC
      - main.ml2db.mulan.learn.crosseval.CrossEvaluator
        - main.ml2db.mulan.learn.traintest.TrainTestEvaluator
          - main.ml2db.mulan.learn.traintest.TrainTestEvaluatorSerialised
  - test.ml2db.al.ExperimentDetailsTest
  - main.ml2db.mulan.learn.Experimenter
  - main.ml2db.mulan.learn.crosseval.Folds
    - main.ml2db.mulan.learn.crosseval.StratifiedFolds
  - main.ml2db.al.utility.globaldistance.GlobalPositionStore
  - main.ml2db.graphviz.GraphViz
    - main.ml2db.graphviz.GraphVizArff
      - main.ml2db.graphviz.GraphVizArffColoured
  - main.ml2db.graphviz.GraphvizInstance
  - main.ml2db.graphviz.GraphVizPrinter
  - main.ml2db.weka.ImportArff
    - main.ml2db.mulan.mulanarff.ImportMulanArff
  - uk.ac.standrews.utils.main.Initialised (implements uk.ac.standrews.utils.main.Initialisable)
    - main.ml2db.al.ActiveLearning
    - main.ml2db.weka.Arff
      - main.ml2db.mulan.mulanarff.MulanArff
        - main.ml2db.mulan.attributesfiltered.AttFiltArff
    - main.ml2db.weka.ArffProps
      - main.ml2db.weka.ArffPropsFromFile
        - main.ml2db.mulan.mulanarff.MulanArffProperties
    - main.ml2db.mulan.attributesfiltered.AttFilter
    - main.ml2db.mulan.learn.crosseval.CrossOutputManager
      - main.ml2db.mulan.learn.traintest.TrainTestOutputManager
        - main.ml2db.al.learning.AlOutputManager
    - main.ml2db.al.learning.ExperimentDetails
    - main.ml2db.mulan.mulanarff.database.MulanArffRecord
  - test.ml2db.InputAndOutputDbManagersTest
  - main.ml2db.weka.InstancesFiller
    - main.ml2db.mulan.mulanarff.MulanInstancesFiller
  - main.ml2db.mulan.mulanarff.InstanceUtils
  - main.ml2db.weka.JaccardDistanceStored (implements weka.core.DistanceFunction, java.io.Serializable)
  - main.ml2db.weka.JaccardDistanceTest
  - test.ml2db.dataharness.LoadFirstValidArff
  - uk.ac.standrews.utils.main.database.Manager
    - main.ml2db.weka.DataSetManager
      - main.ml2db.mulan.mulanarff.MulanDataSetManager
  - mulan.evaluation.measure.MeasureBase (implements mulan.evaluation.measure.Measure, java.io.Serializable)
    - mulan.evaluation.measure.BipartitionMeasureBase
      - mulan.evaluation.measure.LabelBasedBipartitionMeasureBase
        - mulan.evaluation.measure.LabelBasedFMeasure
          - mulan.evaluation.measure.MicroFMeasure
            - main.ml2db.mulan.learn.evaluation.MicroFMeasurePrinter
              - main.ml2db.mulan.learn.evaluation.FalseNegatives
              - main.ml2db.mulan.learn.evaluation.FalsePositives
              - main.ml2db.mulan.learn.evaluation.TruePositives
  - main.ml2db.mulan.mulanarff.database.MulanArffInstancesLoader
  - test.ml2db.mulan.predict.MulanEmitPredictions
  - main.ml2db.mulan.learn.MulanLearner (implements java.io.Serializable)
  - main.ml2db.mulan.learn.MulanLearners
  - main.ml2db.mulan.learn.traintest.MulanTrainExample
  - mulan.data.MultiLabelInstances (implements java.io.Serializable)
    - main.ml2db.mulan.mulanarff.IdMultiLabelInstances (implements java.io.Serializable)
  - mulan.classifier.MultiLabelLearnerBase (implements mulan.classifier.MultiLabelLearner, java.io.Serializable, weka.core.TechnicalInformationHandler)
    - mulan.classifier.lazy.MultiLabelKNN
      - main.ml2db.mulan.classifier.lazy.BRkNNVisible
        - main.ml2db.mulan.classifier.lazy.BRkNNFewInstances
  - test.ml2db.al.utility.neighbourhood.NearestNeighboursByFreqUtilityTest
  - weka.core.neighboursearch.NearestNeighbourSearch (implements weka.core.AdditionalMeasureProducer, weka.core.OptionHandler, weka.core.RevisionHandler, java.io.Serializable)
    - weka.core.neighboursearch.LinearNNSearch
      - main.ml2db.al.utility.neighbours.LinearMultiLabelNNSearch
        - main.ml2db.al.utility.neighbours.LinearNNSearchSkipInstanceItself
  - main.ml2db.al.utility.neighbours.NeighbourhoodExperimentDetails
  - main.ml2db.al.utility.neighbours.NeighbourhoodExperimentDetailsSerialised
  - main.ml2db.mulan.mulanarff.NeighboursPrinter
  - main.ml2db.mulan.mulanarff.NeighboursPrinterTest
  - weka.core.NormalizableDistance (implements weka.core.DistanceFunction, weka.core.OptionHandler, weka.core.RevisionHandler, java.io.Serializable)
    - main.ml2db.weka.JaccardDistance (implements java.io.Serializable)
      - main.ml2db.weka.JaccardEuclideanDistance
  - java.io.OutputStream (implements java.io.Closeable, java.io.Flushable)
    - java.io.ObjectOutputStream (implements java.io.ObjectOutput, java.io.ObjectStreamConstants)
      - main.ml2db.DebuggingObjectOutputStream
  - main.ml2db.mulan.measuremetrics.PredictionsPerformanceMeasurer
  - main.ml2db.mulan.predict.PredictionsSaver
  - main.ml2db.ProjectParameters
    - test.ml2db.dataharness.TestProjectParameters
  - main.ml2db.mulan.predict.QuickPredictor
  - main.ml2db.Serializer
    - main.ml2db.mulan.learn.MulanSerializer
  - main.ml2db.SerializerHelper
  - main.ml2db.mulan.mulanarff.Statistics (implements java.io.Serializable)
  - test.ml2db.mulan.mulanarff.StatisticsTest
  - test.ml2db.al.StatisticsTest
  - uk.ac.standrews.utils.main.database.Table
    - main.ml2db.al.database.AlEvaluationTable
    - main.ml2db.al.database.AlExperimentsTable
    - main.ml2db.weka.ArffRecordsTable
    - main.ml2db.mulan.learn.crosseval.EvaluationResultsTable
    - main.ml2db.mulan.predict.PredictionsExperimentTable
    - main.ml2db.mulan.predict.PredictionsRecordTable
    - main.ml2db.al.database.QueryTable
    - main.ml2db.al.database.UtilityTable
  - main.ml2db.mulan.learn.traintest.TrainTestFullRun
  - main.ml2db.al.utility.UtilityMetric
    - main.ml2db.al.utility.confidence.ConfidenceMetric
    - main.ml2db.al.utility.globaldistance.GlobalDistanceMetric
      - main.ml2db.al.utility.hybrid.GlobalDistanceWithConfidenceMetric
    - main.ml2db.al.utility.neighbours.NeighbourhoodNetwork (implements java.io.Serializable)
    - main.ml2db.al.utility.pickers.RandomPicker (implements main.ml2db.al.utility.pickers.Picker)
      - main.ml2db.al.utility.pickers.byfrequency.ByFrequencyPicker
        - main.ml2db.al.utility.pickers.byfrequency.AttributesByFrequencyPicker
        - main.ml2db.al.utility.pickers.byfrequency.AttSetByFrequencyPicker
        - main.ml2db.al.utility.pickers.byfrequency.LabelsByFreqPicker
        - main.ml2db.al.utility.pickers.byfrequency.NearestNeighboursByFreqUtility
      - main.ml2db.al.utility.pickers.LabelRandomPicker
      - main.ml2db.al.utility.pickers.RandomAttributePicker
      - main.ml2db.al.utility.pickers.RandomAttributeSetPicker
      - main.ml2db.al.utility.pickers.RandomInstancePicker
      - main.ml2db.al.utility.pickers.RandomLabelsPicker
      - main.ml2db.al.utility.pickers.RandomNeigbourhoodPicker

## Interface Hierarchy

- main.ml2db.al.utility.pickers.Picker

## Enum Hierarchy

- java.lang.Object
  - java.lang.Enum<E> (implements java.lang.Comparable<T>, java.io.Serializable)
    - main.ml2db.mulan.classifier.lazy.BRkNNVisible.ExtensionType

- Overview
- Package
- Class
- Use
- Tree
- Deprecated
- Index
- Help

- Prev
- Next

- Frames
- No Frames

- All Classes
